# Ejection fraction and absolute delta troponin in the early emergency department assessment of high coronary disease burden in NSTEMI patients: a retrospective observational study

**DOI:** 10.1186/s12873-026-01690-w

**Published:** 2026-07-16

**Authors:** Remzi Çetinkaya, Mehmet Özel, Songül Doğan Araç, İsmail Başaslan, MehmetSait Coşkun

**Affiliations:** 1 Department of Emergency Medicine, University of Health Sciences, Diyarbakır Gazi Yaşargil Training and Research Hospital, Diyarbakır, Turkey; 2 Department of Cardiology, Ergani State Hospital, Diyarbakır, Turkey

**Keywords:** non-ST-segment elevation myocardial infarction, Emergency department, Gensini score, Ejection fraction, Absolute delta troponin

## Abstract

**Background:**

Patients with non-ST-segment elevation myocardial infarction (NSTEMI) are commonly evaluated first in the emergency department, where early estimation of coronary disease burden may support clinical risk assessment before invasive coronary angiography. This study investigated whether routinely available emergency department parameters, with particular emphasis on ejection fraction (EF) and absolute delta troponin, were associated with high angiographic coronary disease burden assessed by the Gensini score.

**Methods:**

This retrospective observational study included adult NSTEMI patients admitted to the emergency department of a tertiary care hospital between January 1, 2024, and May 1, 2025, who subsequently underwent coronary angiography. Patients were divided into low and high Gensini groups using a predefined threshold of 40. Demographic characteristics, comorbidities, laboratory variables, echocardiographic findings, and absolute changes in serial troponin measurements were compared between groups. Independent associations with the high Gensini category were evaluated using multivariable binary logistic regression. Receiver operating characteristic curve analysis was used to assess the discriminatory performance of EF and absolute delta troponin/100.

**Results:**

A total of 394 patients were included in the final analysis; 209 were classified into the low Gensini group and 185 into the high Gensini group. In the adjusted model, lower EF was independently associated with the high Gensini category (OR: 0.888; 95% CI: 0.859–0.918; *p* < 0.001), while higher absolute delta troponin/100 showed a positive independent association (OR: 1.161; 95% CI: 1.074–1.254; *p* < 0.001). Diabetes mellitus was also independently associated with the high Gensini category (OR: 1.851; 95% CI: 1.096–3.127; *p* = 0.021). EF and absolute delta troponin/100 both showed statistically significant discrimination for the high Gensini category, with AUC values of 0.783 and 0.758, respectively.

**Conclusions:**

In NSTEMI patients evaluated in the emergency department, lower EF and higher absolute delta troponin were independently associated with high angiographic coronary disease burden. EF showed slightly higher discriminatory performance than absolute delta troponin/100. These findings suggest that EF and absolute troponin change may provide complementary information during early emergency department assessment before coronary angiography, although they should not be interpreted as validated predictors of clinical outcomes.

## Background

Acute coronary syndromes continue to rank among the leading causes of cardiovascular morbidity and mortality, despite substantial advances in diagnosis and treatment. Within this spectrum, non-ST-segment elevation myocardial infarction (NSTEMI) requires careful early risk stratification because of its heterogeneous clinical presentation and the difficulty of reliably predicting disease severity [1]. Current guidelines emphasize the importance of integrating clinical findings, electrocardiographic changes, biomarker dynamics, and imaging data when assessing patients with NSTEMI [2, 3].

Accurate assessment of the extent and severity of coronary artery disease in patients with NSTEMI is critically important for both determining the treatment strategy and predicting prognosis. For this purpose, the Gensini score is an established angiographic measure that quantitatively reflects the total atherosclerotic burden by weighting the degree of coronary stenosis according to the functional significance of the anatomical location of the lesion. The scoring system used to calculate the Gensini score is presented in Table 1. Recent studies have shown that a higher Gensini score is associated with adverse clinical outcomes in patients with coronary artery disease and may also have prognostic value for short-term mortality in the acute coronary syndrome population. In this respect, the Gensini score is considered not only a tool for defining angiographic disease extent, but also a parameter that provides a meaningful contribution to clinical risk assessment [4, 5].

**Table 1:** Gensini score calculation system

Degree of stenosis	Stenosis score
≤ 25% stenosis	1
26–50% stenosis	2
51–75% stenosis	4
76–90% stenosis	8
91–99% stenosis	16
100% total occlusion	32
**Coronary segment**	**Segment weighting factor**
Left main coronary artery	5
Proximal left anterior descending artery	2.5
Proximal left circumflex artery	2.5
Mid left anterior descending artery	1.5
Distal left anterior descending artery	1
Right coronary artery	1
Posterior descending artery	1
Posterolateral artery	1
Obtuse marginal artery	1
Other small segments	0.5

In biomarker-based assessment, cardiac troponin is one of the fundamental components of NSTEMI diagnosis; however, its clinical value is not limited to diagnostic confirmation alone. In particular, the absolute change observed in serial measurements, namely absolute delta troponin, may provide additional information regarding underlying significant coronary obstruction by reflecting the dynamic extent of myocardial injury [6]. Similarly, echocardiographic parameters are also important because they demonstrate the impact of coronary disease severity on myocardial function. Left ventricular ejection fraction is the most widely used indicator of global systolic function, and reduced ejection fraction has been shown to be associated with more extensive obstructive coronary artery disease and poorer clinical outcomes. In addition, global hypokinesia reflects diffuse impairment of ventricular contractility, whereas segmental wall motion abnormality indicates regional ischemic involvement, thereby providing complementary information regarding coronary lesion burden [7].

Although clinical, laboratory, and echocardiographic variables are routinely available during the early emergency department assessment of NSTEMI patients, their relationship with high angiographic coronary disease burden remains incompletely defined. In particular, the comparative value of absolute delta troponin and EF for identifying patients with a high Gensini score before invasive coronary angiography has not been sufficiently clarified. Therefore, this study aimed to evaluate the association of readily available emergency department parameters, particularly EF and absolute delta troponin, with high Gensini score in patients with NSTEMI.

## Methods

### Study design and setting

We conducted a retrospective observational study in the emergency department of a tertiary care center. Adult patients presenting between January 1, 2024, and May 1, 2025, with a diagnosis of non-ST-segment elevation myocardial infarction (NSTEMI) were identified through hospital records and included in the retrospective review. All eligible patient encounters occurred before ethics committee approval, and data extraction and analysis were performed retrospectively after approval was obtained. The study focused on clinical, laboratory, and echocardiographic variables available during the early emergency department evaluation before invasive coronary angiography. NSTEMI diagnosis was established by a cardiologist based on the combined assessment of clinical presentation, electrocardiographic findings, and serial troponin measurements.

Coronary artery disease burden was assessed using the Gensini scoring system. The Gensini score was dichotomized using a threshold of 40, which has been used in previous studies to define higher angiographic coronary artery disease burden. However, this threshold was derived from a non-NSTEMI population and has not been validated as an NSTEMI-specific clinical cut-off. Therefore, in the present study, it was used only to define an angiographic disease burden category. This threshold was determined a priori before statistical analyses to avoid data-driven cut-off selection. Accordingly, patients with a Gensini score < 40 were classified as the low Gensini group, whereas those with a Gensini score ≥ 40 were classified as the high Gensini group [8].

The study protocol was approved by the Clinical Research Ethics Committee of the same hospital (Decision No: 517; Date: June 13, 2025). All procedures were conducted in compliance with the Declaration of Helsinki. Since the study was retrospective and based on anonymized patient data, written informed consent was not required.

## Patient selection

Adult patients aged 18 years or older who were diagnosed with NSTEMI in the emergency department, admitted for inpatient follow-up, underwent coronary angiography, had a calculable Gensini score, and had complete clinical, laboratory, echocardiographic, troponin, and angiographic data were included in the study.

Patients with ST-segment elevation myocardial infarction, unstable angina, age younger than 18 years, absence of coronary angiography, non-calculable Gensini score, missing essential laboratory or echocardiographic data, unavailable baseline and/or 3-hour troponin measurements, or recurrent presentations other than the first admission were excluded.

## Data collection and definitions

Patient-level clinical, laboratory, echocardiographic, and angiographic variables were obtained through a retrospective review of the hospital electronic database and medical records. Age and sex were recorded as demographic variables. Diabetes mellitus, hypertension, chronic obstructive pulmonary disease, history of coronary artery disease, and history of coronary artery bypass grafting were recorded as comorbid conditions. The GRACE score was calculated using available clinical, electrocardiographic, laboratory, and presentation-related variables and was included in the descriptive comparison of continuous variables.

Laboratory parameters obtained during the emergency department evaluation were recorded, including glucose, urea, C-reactive protein (CRP), neutrophil-to-lymphocyte ratio (NLR), platelet-to-lymphocyte ratio (PLR), HALP score, and lymphocyte-to-CRP ratio. HALP score was calculated as hemoglobin × albumin × lymphocyte count / platelet count.

Serial troponin measurements were performed according to the routine emergency department protocol, with blood samples obtained at admission and approximately 3 h later. Only patients with both baseline and 3-hour troponin values available were included. Troponin was measured in the central laboratory using the high-sensitivity cardiac troponin T assay on a Cobas E411 analyzer, and the laboratory upper reference limit was accepted as 14 ng/L. Absolute delta troponin was calculated as the 3-hour troponin value minus the baseline troponin value and was expressed in ng/L. Patients with a lower 3-hour troponin value than the baseline value were retained in the analysis with negative absolute delta troponin values. In the multivariable regression and ROC analyses, absolute delta troponin was divided by 100 to facilitate clinical interpretation of the regression coefficient, odds ratio, and cut-off value.

### Echocardiographic assessment

Transthoracic echocardiographic assessment was performed in the emergency department by a cardiologist before coronary angiography. EF was assessed by visual estimation by the attending cardiologist during routine emergency department echocardiographic evaluation. Global hypokinesia and segmental wall motion abnormality were recorded from routine echocardiography reports. EF was evaluated as a continuous variable, whereas global hypokinesia and segmental wall motion abnormality were recorded as binary variables according to their presence or absence.

### Gensini score assessment

For each patient, coronary angiography reports and images were reviewed, and the Gensini score was calculated by a cardiologist. The Gensini scoring system assigns severity scores according to the degree of luminal stenosis and multiplies these scores by weighting factors based on the anatomical importance of the involved coronary segment. The scoring system used in this study is presented in Table 1.

Patients were then categorized into low and high Gensini groups according to the predefined threshold of 40. In all analyses, the dependent variable was membership in the high Gensini category.

### Statistical analysis

All statistical analyses were performed with IBM SPSS Statistics for Windows, version 27.0 (IBM Corp., Armonk, NY, USA). Patients were stratified according to their Gensini scores into low and high Gensini groups, and the high Gensini category was used as the dependent outcome variable in the analyses.

Categorical variables were reported as n (%), and group differences were assessed using Pearson’s chi-square test or Fisher’s exact test, depending on expected cell frequencies. Continuous variables were first examined for distributional properties. Variables that did not follow a normal distribution were presented as median (Q1–Q3), and comparisons between the two groups were performed using the Mann–Whitney U test.

Multivariable binary logistic regression analysis was used to determine independent factors associated with classification into the high Gensini category. Variable selection for the final model was guided by clinical relevance, model parsimony, and assessment of multicollinearity rather than by univariable statistical significance alone. Age, sex, diabetes mellitus, hypertension, EF, and absolute delta troponin/100 were included in the final model. Before constructing the final multivariable logistic regression model, multicollinearity among the variables included in the model was assessed using tolerance and variance inflation factor (VIF) values obtained from collinearity diagnostics. A VIF value greater than 5 and a tolerance value below 0.20 were considered indicative of potentially relevant multicollinearity.

Results were reported as regression coefficient (B), odds ratio [OR, Exp(B)], 95% confidence interval (CI), and p value. The fit of the logistic regression model was assessed using the Omnibus test, Hosmer–Lemeshow goodness-of-fit test, Nagelkerke R² value, and overall classification accuracy.

To address the potential information loss related to dichotomization of the Gensini score, an additional multiple linear regression analysis was performed using the continuous Gensini score as the dependent variable. Because the Gensini score showed a right-skewed distribution, it was log-transformed as ln(Gensini score + 1) before linear regression analysis. The same covariates used in the primary logistic regression model, including age, sex, diabetes mellitus, hypertension, EF, and absolute delta troponin/100, were included in this additional model.

The discriminatory performance of absolute delta troponin/100 and EF for identifying the high Gensini category was evaluated using receiver operating characteristic curve analysis. The area under the curve, 95% confidence interval, optimal threshold, sensitivity, and specificity were calculated for both parameters. Since increasing absolute delta troponin/100 values reflected a greater probability of high Gensini classification, the ROC analysis was conducted directly for this variable. For EF, because lower values were associated with the high Gensini category, the transformation 100 − EF was used in the ROC analysis to ensure directional consistency. However, the results were re-expressed using the EF cut-off value to facilitate clinical interpretation.

## Results

**Fig. 1:**
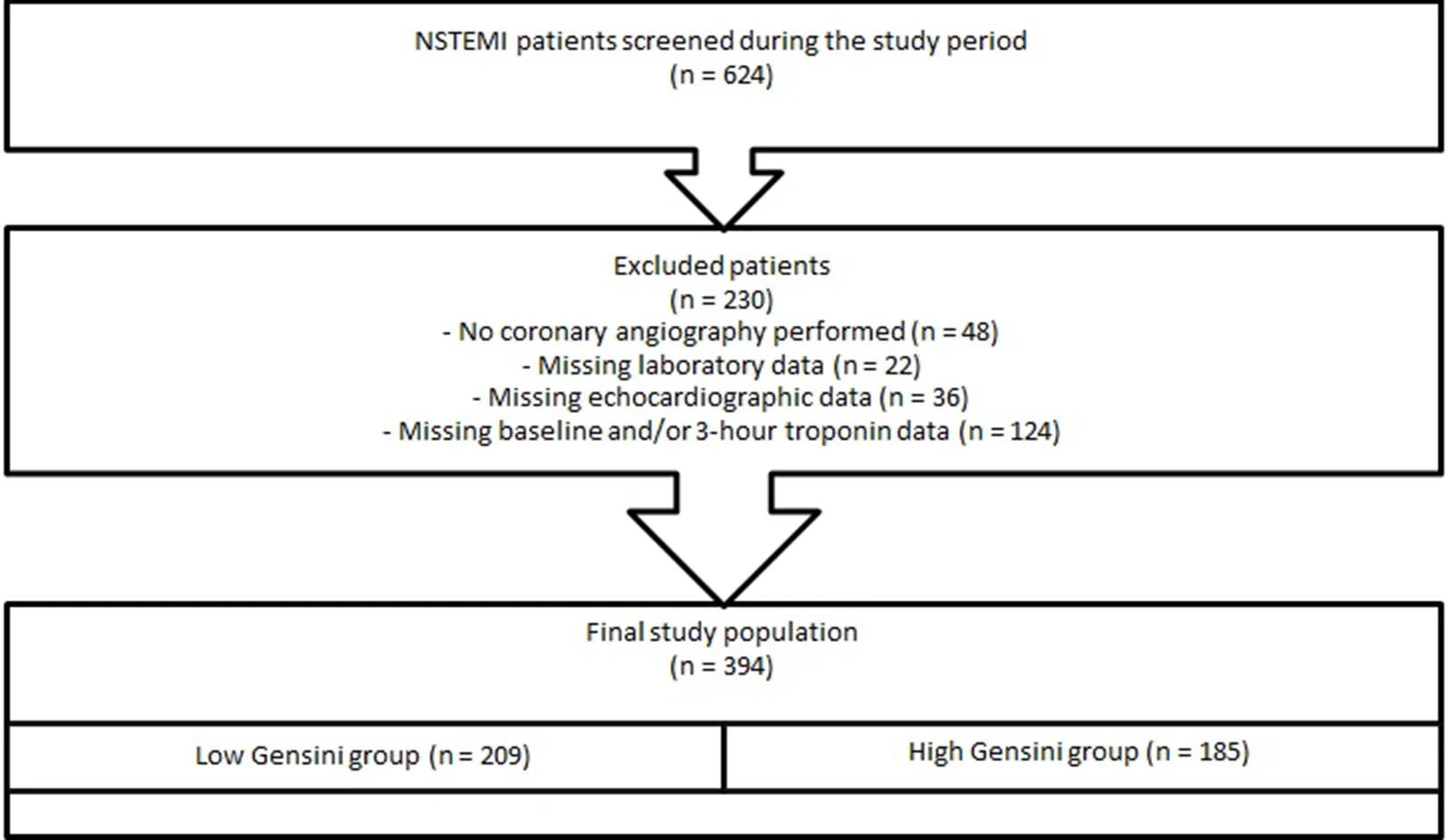
Flow diagram of patient selection and group allocation

During the study period, 624 patients with NSTEMI were screened for eligibility. Of these, 230 patients were excluded because coronary angiography was not performed (*n* = 48), laboratory data were missing (*n* = 22), echocardiographic data were missing (*n* = 36), or baseline and/or 3-hour troponin data were unavailable (*n* = 124). Therefore, 394 patients were included in the final analysis. According to the predefined Gensini score threshold, 209 patients were classified into the low Gensini group and 185 patients into the high Gensini group (Fig. 1).

There was no significant between-group difference in sex distribution, as reflected by the proportion of female patients (*p* = 0.768). Chronic obstructive pulmonary disease was also comparable between the two groups (*p* = 0.083). By contrast, a history of coronary artery bypass grafting was significantly more frequent in the high Gensini group (*p* = 0.034). Patients in the high Gensini group also had significantly higher rates of diabetes mellitus, hypertension, and previous coronary artery disease (*p* < 0.001, *p* < 0.001, and *p* = 0.009, respectively). Echocardiographic assessment demonstrated that global hypokinesia and segmental wall motion abnormality were both more commonly detected in the high Gensini group than in the low Gensini group (*p* < 0.001 for both) (Table 2).

**Table 2:** Comparison of categorical variables between the low and high Gensini groups

Variable	Low Gensini (*n* = 209), n (%)	High Gensini (*n* = 185), n (%)	*p*
Female sex	66 (31.6)	61 (33.0)	0.768
History of CABG	6 (2.9)	14 (7.6)	0.034
Diabetes mellitus	75 (35.9)	109 (58.9)	< 0.001
Hypertension	97 (46.4)	118 (63.8)	< 0.001
COPD	15 (7.2)	6 (3.2)	0.083
History of coronary artery disease	69 (33.0)	85 (45.9)	0.009
Global hypokinesia	19 (9.1)	97 (52.4)	< 0.001
Segmental wall motion abnormality	21 (10.0)	46 (24.9)	< 0.001

CABG: Coronary Artery Bypass Grafting, COPD: Chronic Obstructive Pulmonary Disease

Continuous variable analysis demonstrated a distinct clinical and laboratory profile in patients with high Gensini scores. Compared with the low Gensini group, these patients had higher median age [67 (59–75) vs. 61 (52–70), *p* < 0.001], glucose [140 (106–205) vs. 117 (99–182), *p* < 0.001], urea [39 (31–53) vs. 35 (28–43), *p* < 0.001], CRP [6.40 (2.70–13.50) vs. 4.70 (2.00–8.90), *p* = 0.004], absolute delta troponin [217.00 (85.76–570.00) vs. 46.00 (14.00–127.80) ng/L, *p* < 0.001], and NLR [3.09 (2.03–5.29) vs. 2.62 (1.78–3.98), *p* = 0.023]. The GRACE score was also significantly higher in the high Gensini group than in the low Gensini group [152 (130–177) vs. 119 (97–147), *p* < 0.001]. By contrast, markers reflecting cardiac function and systemic inflammatory–nutritional status were lower in the high Gensini group. Specifically, EF [45 (40–55) vs. 60 (55–60), *p* < 0.001], HALP score [4.72 (2.85–6.66) vs. 5.22 (3.67–7.74), *p* = 0.009], and lymphocyte-to-CRP ratio [0.32 (0.14–0.79) vs. 0.53 (0.25–1.05), *p* < 0.001] were significantly decreased. PLR was higher in the high Gensini group; however, this difference did not reach statistical significance [118.99 (82.70–166.26) vs. 107.27 (77.36–136.02), *p* = 0.052] (Table 3).

**Table 3:** Comparison of continuous variables between the low and high Gensini groups

Variable	Low Gensini (*n* = 209)	High Gensini (*n* = 185)	*p*
Age	61 (52–70)	67 (59–75)	< 0.001
Glucose (mg/dL)	117 (99–182)	140 (106–205)	< 0.001
Urea (mg/dL)	35 (28–43)	39 (31–53)	< 0.001
CRP	4.70 (2.00–8.90)	6.40 (2.70–13.50)	0.004
EF	60 (55–60)	45 (40–55)	< 0.001
Absolute delta troponin, ng/L	46.00 (14.00–127.80)	217.00 (85.76–570.00)	< 0.001
NLR	2.62 (1.78–3.98)	3.09 (2.03–5.29)	0.023
PLR	107.27 (77.36–136.02)	118.99 (82.70–166.26)	0.052
HALP	5.22 (3.67–7.74)	4.72 (2.85–6.66)	0.009
LYM/CRP	0.53 (0.25–1.05)	0.32 (0.14–0.79)	< 0.001
GRACE score	119 (97–147)	152 (130–177)	< 0.001

Data are presented as median (Q1–Q3). Groups were compared using the Mann–Whitney U test. CRP: C-reactive protein; EF: ejection fraction; NLR: neutrophil-to-lymphocyte ratio; PLR: platelet-to-lymphocyte ratio; HALP: hemoglobin, albumin, lymphocyte, and platelet score; LYM/CRP: lymphocyte-to-C-reactive protein ratio

To identify factors independently related to high coronary artery disease burden, a multivariable binary logistic regression model was constructed using the high Gensini category as the dependent variable. The model showed overall statistical significance (Omnibus test *p* < 0.001), adequate goodness of fit (Hosmer–Lemeshow *p* = 0.264), a Nagelkerke R² value of 0.395, and an overall classification accuracy of 76.9%. Collinearity diagnostics did not show problematic multicollinearity among the variables included in the multivariable model. The VIF values ranged from 1.075 to 1.321, and tolerance values ranged from 0.757 to 0.930. These findings indicated that the independent variables could be entered into the model without relevant multicollinearity. After adjustment, diabetes mellitus, EF, and absolute delta troponin/100 were independently associated with the high Gensini category. Diabetes mellitus was associated with higher odds of belonging to the high Gensini group (OR: 1.851; 95% CI: 1.096–3.127; *p* = 0.021). Higher EF was inversely associated with high Gensini classification, with each 1-unit increase in EF reducing the odds of belonging to the high Gensini group (OR: 0.888; 95% CI: 0.859–0.918; *p* < 0.001). In contrast, absolute delta troponin/100 showed a positive independent association, indicating that each 100-ng/L increase in absolute delta troponin was related to higher odds of high Gensini classification (OR: 1.161; 95% CI: 1.074–1.254; *p* < 0.001). No independent associations were observed for age, hypertension, or sex in the adjusted model (Table 4).

**Table 4:** Variables independently associated with the high Gensini category: multivariable binary logistic regression analysis

Variable	B	OR (Exp(B))	95% CI	*p*
Age	0.009	1.009	0.987–1.032	0.425
DM	0.616	1.851	1.096–3.127	0.021
HT	0.148	1.159	0.678–1.981	0.589
Sex	-0.018	0.982	0.575–1.679	0.948
EF	-0.119	0.888	0.859–0.918	< 0.001
Absolute delta troponin/100	0.149	1.161	1.074–1.254	< 0.001

B indicates the logistic regression coefficient; OR [Exp(B)], odds ratio; 95% CI, 95% confidence interval for the odds ratio; and p, p value. The positive outcome was defined as membership in the high Gensini category. Absolute delta troponin was calculated as the 3-hour troponin value minus the baseline troponin value and was divided by 100 before being entered into the model to facilitate clinical interpretation. DM: Diabetes mellitus, HT: Hypertension, EF: Ejection fraction

To evaluate whether the associations remained consistent when the Gensini score was treated as a continuous variable, an additional multiple linear regression analysis was performed using ln(Gensini score + 1) as the dependent variable. The model was statistically significant (F = 30.908; *p* < 0.001), with an R² value of 0.324 and an adjusted R² value of 0.313. In this model, EF remained inversely associated with continuous Gensini score (B: -0.059; 95% CI: -0.070 to -0.047; *p* < 0.001), while absolute delta troponin/100 remained positively associated with continuous Gensini score (B: 0.022; 95% CI: 0.009 to 0.036; *p* = 0.002). Sex was also associated with continuous Gensini score (B: 0.277; 95% CI: 0.084 to 0.469; *p* = 0.005), whereas age, diabetes mellitus, and hypertension were not independently associated in this additional model. These findings indicate that the associations of EF and absolute delta troponin/100 were preserved when angiographic disease burden was analyzed across the continuous Gensini score spectrum.

**Fig. 2:**
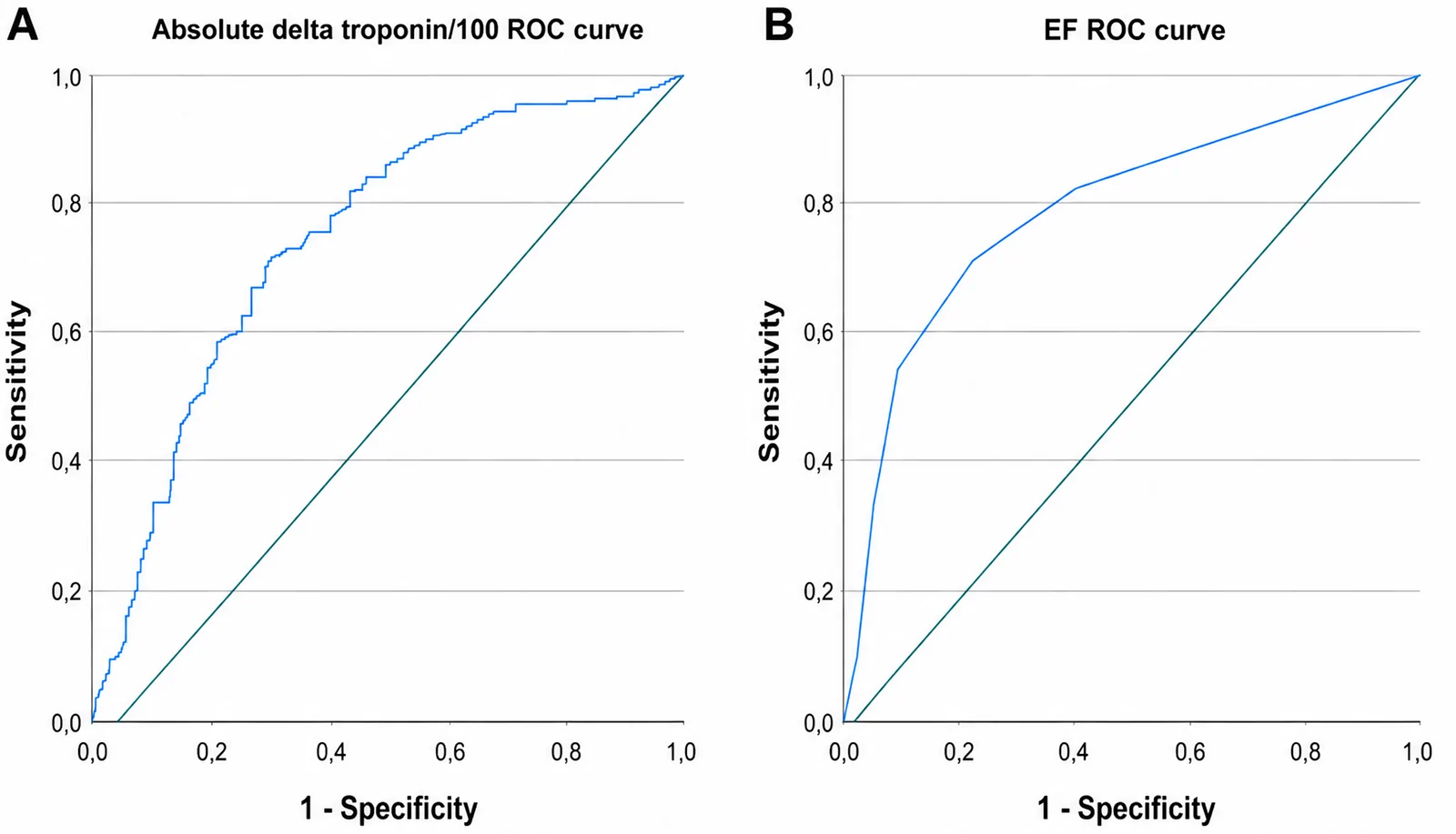
ROC curves for absolute delta troponin/100 and EF in discriminating the high Gensini category. **A**: Absolute delta troponin/100 demonstrated an AUC of 0.758 (95% CI: 0.710–0.805; p < 0.001), with an optimal cut-off value of 1.100, sensitivity of 69.7%, and specificity of 72.2%. **B**: EF demonstrated an AUC of 0.783 (95% CI: 0.736–0.830; p < 0.001), with an optimal cut-off value of EF ≤ 52.5%, sensitivity of 71.4%, and specificity of 77.0%. For EF, ROC analysis was performed using the 100 − EF transformation because lower EF values were associated with the high Gensini category

ROC analysis was performed for absolute delta troponin/100 and EF to evaluate their performance in discriminating the high Gensini category. For absolute delta troponin/100, the area under the curve (AUC) was 0.758 (95% CI: 0.710–0.805; *p* < 0.001), and the optimal cut-off value was determined as 1.100. At this threshold, sensitivity was 69.7% and specificity was 72.2%. For EF, because lower values were associated with the high Gensini category, the transformation 100 − EF was used in the ROC analysis; however, the results were re-expressed in terms of EF for easier clinical interpretation. Accordingly, the AUC for EF was 0.783 (95% CI: 0.736–0.830; *p* < 0.001), and the optimal cut-off value was approximately EF ≤ 52.5%; at this point, sensitivity was 71.4% and specificity was 77.0%. These findings indicate that EF and absolute delta troponin/100 both showed statistically significant discrimination for the high Gensini category, with EF demonstrating slightly higher discriminatory performance (Table 5; Fig. 2).

**Table 5:** ROC analysis of absolute delta troponin/100 and EF in discriminating the high Gensini category

Variable	AUC	95% CI	Cut-off	Sensitivity (%)	Specificity (%)	*p*
Absolute delta troponin/100	0.758	0.710–0.805	1.100	69.7	72.2	< 0.001
EF*	0.783	0.736–0.830	≤ 52.5	71.4	77.0	< 0.001

*For EF, ROC analysis was performed using the 100 − EF transformation to evaluate the association between lower EF values and the high Gensini category in a positive direction. EF: Ejection fraction

## Discussion

The principal finding of this study was that lower EF and higher absolute delta troponin were independently associated with high angiographic coronary disease burden in NSTEMI patients evaluated in the emergency department. In addition, diabetes mellitus remained independently associated with the high Gensini category in the adjusted model. Both EF and absolute delta troponin/100 showed statistically significant discriminatory performance for identifying the high Gensini category, with EF demonstrating slightly higher discrimination. These findings suggest that early echocardiographic assessment and absolute changes in serial troponin measurements may provide complementary information regarding the likelihood of increased coronary disease burden before invasive coronary angiography.

The inverse independent association between EF and the high Gensini category suggests that impairment of left ventricular systolic function may coexist with a more extensive coronary atherosclerotic burden. This finding is consistent with studies reporting an association between echocardiographic functional parameters and angiographic disease severity in patients with NSTEMI [9, 10]. In the present study, EF showed slightly higher discriminatory performance than absolute delta troponin/100 for identifying the high Gensini category. This finding supports the clinical relevance of early echocardiographic evaluation, not as a replacement for angiography or guideline-based risk stratification, but as a practical functional marker that may reflect the myocardial impact of underlying coronary disease burden. Previous studies have also reported that reduced EF is associated with adverse clinical outcomes in patients with NSTEMI [9, 11, 12]; however, the present study evaluated angiographic disease burden rather than clinical outcomes.

Absolute delta troponin/100 was also independently associated with the high Gensini category. This finding is biologically plausible because absolute changes in serial high-sensitivity troponin measurements reflect the dynamic extent of myocardial injury. Kim et al. reported that the 0–3-hour change in high-sensitivity troponin T was associated with significant coronary stenosis in patients with acute coronary syndrome, and that the rate of significant stenosis was higher among patients with a positive interval change [6]. Similarly, Sanchis et al. demonstrated that maximum and delta hs-cTnT values were associated with coronary angiographic findings in patients with non-ST-elevation acute chest pain [13]. In contrast, Bravo et al. reported that hs-cTnI levels were associated with stenosis severity in patients with NSTEMI, but that their performance in discriminating severe coronary disease remained limited [14]. Our findings are consistent with this framework and suggest that absolute delta troponin/100 is associated with the high Gensini category. Although absolute delta troponin/100 showed statistically significant discriminatory performance, it should be interpreted as a complementary marker rather than a standalone clinical decision tool. In the present analysis, the discriminatory performance of absolute delta troponin/100 was statistically significant and only slightly lower than that of EF.

Diabetes mellitus, hypertension, history of coronary artery disease, and history of CABG were more frequent in the high Gensini group, suggesting that high angiographic coronary disease burden often develops on a background of pre-existing diffuse atherosclerosis. This finding is consistent with previous reports showing that classical cardiovascular risk factors, including diabetes mellitus, are associated with greater angiographic severity of coronary artery disease as assessed by the Gensini score [15]. In the adjusted model, diabetes mellitus remained independently associated with the high Gensini category, whereas age, hypertension, and sex did not show independent associations. This finding is also consistent with the established role of diabetes mellitus in the development of diffuse and more severe coronary atherosclerosis. In NSTEMI patients, diabetes may contribute to more extensive coronary involvement through endothelial dysfunction, chronic inflammation, accelerated atherosclerosis, and microvascular impairment. Therefore, the independent association between diabetes mellitus and the high Gensini category supports the view that chronic cardiometabolic risk factors, together with acute myocardial injury markers and left ventricular systolic function, contribute to angiographic coronary disease burden.

The distribution of echocardiographic findings also supports this interpretation. The higher frequency of global hypokinesia and segmental wall motion abnormality in the high Gensini group indicates that severe coronary disease burden is more prominently reflected in myocardial function. Previous studies have reported that segmental wall motion abnormalities can independently predict obstructive coronary artery disease in patients with acute chest pain and are associated with a more adverse clinical course in patients with NSTEMI [16]. Therefore, the echocardiographic findings in our study support the view that the high Gensini category reflects not only angiographic disease extent, but also functional cardiac involvement.

When clinical and biochemical parameters were evaluated, patients in the high Gensini group had higher age, glucose, urea, CRP, NLR, and absolute delta troponin values, together with lower EF, HALP score, and lymphocyte-to-CRP ratio. These findings suggest that greater angiographic coronary disease burden may coexist with more pronounced metabolic dysregulation, inflammatory activation, myocardial injury, and impaired inflammatory–nutritional status. In the NSTEMI population, advanced age has been associated with higher comorbidity burden and worse short-term outcomes, while elevated glucose levels have been linked to more severe coronary artery disease [17, 18]. Similarly, CRP has been reported to correlate positively with the severity of coronary stenosis, and NLR has been shown to increase in patients with more severe coronary artery disease [19, 20]. Regarding the lymphocyte-to-CRP ratio, a previous study demonstrated that a lower ratio may be associated with greater coronary artery disease severity, which is consistent with our findings [21]. The lower HALP score observed in the high Gensini group may also reflect impairment in the inflammation–nutrition axis. Recent studies have reported that a low HALP score is associated with adverse outcomes in patients with NSTEMI [22]. However, these laboratory and inflammatory parameters should be interpreted as descriptive group-level differences rather than independent determinants, because they were not included simultaneously in the final multivariable model.

The higher GRACE score in the high Gensini group supports the clinical consistency of our findings. Although this study evaluated angiographic disease burden rather than clinical outcomes, patients with higher Gensini scores also had a higher guideline-based clinical risk profile, consistent with contemporary evidence supporting GRACE-based risk assessment in NSTE-ACS [23].

From an emergency medicine perspective, these findings suggest that EF and absolute delta troponin may provide practical complementary information during the early assessment of NSTEMI patients before invasive coronary angiography. However, these parameters should not be interpreted as standalone triage tools or as substitutes for established risk scores, guideline-based invasive strategy decisions, or angiographic evaluation. Rather, they may help characterize patients with a higher likelihood of increased angiographic coronary disease burden during the initial emergency department evaluation. Because the outcome of this study was an angiographic disease burden category rather than a clinical endpoint, the findings should not be extrapolated to mortality, recurrent myocardial infarction, revascularization outcomes, or major adverse cardiovascular events without further prospective validation.

## Limitations

This study has several limitations. First, this was a retrospective single-center study, which may limit the generalizability of the findings and introduce selection bias. In particular, the exclusion of patients without both baseline and 3-hour troponin measurements represents an additional source of potential selection bias. Because absolute delta troponin could only be calculated in patients with available serial troponin data, 124 patients were excluded for missing baseline and/or 3-hour troponin values. These excluded patients may not have been missing at random. For example, patients who underwent very early invasive management, were transferred, were discharged early, or had a different clinical trajectory may have been less likely to have complete serial troponin sampling. Therefore, the final study population may not fully represent the entire spectrum of NSTEMI patients presenting to the emergency department, and the observed associations between absolute delta troponin, EF, and the high Gensini category should be interpreted with this potential selection bias in mind.

Second, the primary outcome of this study was an angiographic disease burden category based on the Gensini score rather than a clinical endpoint. Although the Gensini score provides a quantitative assessment of coronary artery disease severity, it does not directly represent patient-centered outcomes such as mortality, recurrent myocardial infarction, heart failure, urgent revascularization, or major adverse cardiovascular events. Therefore, the present findings should not be interpreted as evidence that EF or absolute delta troponin predicts clinical outcomes. In addition, the Gensini score primarily reflects angiographically visible epicardial coronary stenosis and does not adequately capture small-vessel coronary disease or microvascular dysfunction. MINOCA was not evaluated as a separate subgroup in the present study, because its diagnosis requires additional clinical, imaging, and etiological assessment beyond the angiographic Gensini score. Therefore, patients with non-obstructive coronary arteries, if present, would have been classified within the low Gensini category rather than analyzed as a distinct MINOCA population.

Third, the Gensini score was dichotomized using a predefined threshold of 40. Although this threshold has been used in previous studies, it was derived from a non-NSTEMI population and should not be interpreted as a validated NSTEMI-specific clinical cut-off. Dichotomization of a continuous angiographic score may lead to loss of information and may obscure relationships across the full spectrum of disease severity. To address this limitation, we performed an additional multiple linear regression analysis using the continuous log-transformed Gensini score; however, the primary analysis still relied on a categorical high Gensini definition.

Fourth, only variables available in the retrospective medical records were included in the analysis. Potentially relevant anatomical and treatment-related factors, including symptom duration, culprit lesion characteristics, number of diseased vessels, lesion complexity, medication use, exact timing of invasive management, and revascularization strategy, could not be fully evaluated as separate variables. These unmeasured factors may have influenced EF, troponin dynamics, and angiographic disease burden. In addition, EF was assessed by visual estimation during routine emergency department echocardiography, which may have introduced measurement variability compared with standardized quantitative methods such as the modified Simpson method. Furthermore, formal interobserver or intraobserver variability analysis for the Gensini score calculation was not performed. Although Gensini scores were calculated by a cardiologist using coronary angiography reports and images, the absence of reproducibility assessment may have introduced measurement variability in angiographic disease burden classification.

Finally, although EF and absolute delta troponin/100 were independently associated with the high Gensini category, their use should be considered complementary rather than definitive. The discriminatory performance of these parameters does not support their use as standalone clinical decision tools. Prospective multicenter studies including standardized serial troponin sampling, detailed angiographic variables, and clinical outcome data are needed to validate these findings.

## Conclusion

In NSTEMI patients evaluated in the emergency department, lower EF and higher absolute delta troponin were independently associated with high angiographic coronary disease burden as assessed by the Gensini score. Diabetes mellitus was also independently associated with the high Gensini category. EF and absolute delta troponin/100 both showed statistically significant discriminatory performance, with EF demonstrating slightly higher discrimination. These findings suggest that EF and absolute troponin change may provide complementary information during early emergency department assessment before invasive coronary angiography; however, they should not be interpreted as validated predictors of clinical outcomes or as parameters that directly determine therapeutic strategy or the timing of invasive management. Further prospective multicenter studies including clinical outcome data are needed to confirm these findings.

## Data Availability

The datasets used and/or analysed during the current study are available from the corresponding author on reasonable request, subject to institutional and ethical restrictions.
